# Quasisolitons in self-diffusive excitable systems, or Why asymmetric diffusivity obeys the Second Law

**DOI:** 10.1038/srep30879

**Published:** 2016-08-05

**Authors:** V. N. Biktashev, M. A. Tsyganov

**Affiliations:** 1 College of Engineering, Mathematics and Physical Sciences, University of Exeter, Exeter EX4 4QF, UK; 2 EPSRC Centre for Predictive Modelling in Healthcare, University of Exeter, Exeter, EX4 4QJ, UK; 3 Institute of Theoretical and Experimental Biophysics, Pushchino, Moscow Region, 142290, Russia

## Abstract

Solitons, defined as nonlinear waves which can reflect from boundaries or transmit through each other, are found in conservative, fully integrable systems. Similar phenomena, dubbed quasi-solitons, have been observed also in dissipative, “excitable” systems, either at finely tuned parameters (near a bifurcation) or in systems with cross-diffusion. Here we demonstrate that quasi-solitons can be robustly observed in excitable systems with excitable kinetics and with self-diffusion only. This includes quasi-solitons of fixed shape (like KdV solitons) or envelope quasi-solitons (like NLS solitons). This can happen in systems with more than two components, and can be explained by effective cross-diffusion, which emerges via adiabatic elimination of a fast but diffusing component. We describe here a reduction procedure can be used for the search of complicated wave regimes in multi-component, stiff systems by studying simplified, soft systems.

## Motivation

Reaction-diffusion systems are an important mathematical tool in studying dissipative structures. For the properties of solutions of these systems, diffusion is as important as is reaction. For instance, Turing structures are possible only if there is significant difference in the diffusion coefficients between activator and inhibitor species^1^. Most of literature on waves and patterns is focussed on self-diffusion, when the flux of a reacting species is defined by the gradient of that same species. However, this ignores the phenomenon of cross-diffusion, when the flux of one species depends on gradient of another species.

There is a large variety of interesting regimes in systems with nonlinear kinetics and cross-diffusion instead of or in additional to self-diffusion^2^. Some of these regimes present a considerable theoretical interest, since they manifest properties that are traditionally associated with very different “realms”: on one hand, these are waves that preserve stable and often unique profile, speed and amplitude, similar to nerve pulse, on the other hand, they can penetrate through each other or reflect from boundaries, such as waves in linear systems or solitons in conservative nonlinear systems. It is interesting where in nature such regimes can be observed.

Originally, these regimes have been discovered in models motivated by population dynamics, where (nonlinear) cross-diffusion appears naturally as taxis of micro- or macro-organisms with respect to each other’s population density^3^,^4^. There are also examples of similar models occurring in physical applications, see e.g. ref. 5. However, real cross-diffusion is actually observed in chemical solutions along with the self-diffusion^6^, which brings up the question of the possibility of observing the quasi-solitons in excitable chemical reactions, such as BZ reaction or some of its modifications, which would be a convenient experimental object to observe quasi-solitons.

This question encounters a number of obstacles due to physical constraints. Firstly, the cross-diffusion must always be nonlinear in order to preserve positivity of chemical concentrations^7^. Then, there is a fundamental requirement that the diffusivity matrix should have all real and positive eigenvalues^8^, which follows from Onsager reciprocal relations^9^,^10^ based on the Second Law of Thermodynamics. The first of these obstacles does not seem particularly essential: quasi-solitons have been observed in models both with linear^2^,^11^,^12^ and non-linear^3^,^4^ cross-diffusion. The second obstacle is more serious, as in all the known theoretical examples, the diffusivity matrix required for observation of quasi-solitons, has zero or complex eigenvalues. Hence, as argued e.g. in [ref. 6, page 899], it would appear that there are no chances to observe these regimes in chemical excitable systems.

In the present paper, we seek to look into this question a bit deeper. We are led by the observation that the above fundamental requirement is about actual diffusivities, which would appear in reactions written in all their elementary steps, for all intermediate reacting species, whereas for studies of waves and pattern formation one typically uses reduced mathematical models, and the diffusivities in them are *effective*. Pattern-forming reactions are supposed to be supported by a source of energy, say in the form of constant supply of a reagent which is consumed in the reaction, whereas the Second Law applies to closed systems. Although this source of energy (and of “negative entropy”, in Schrödinger’s sense^13^) is in the reaction part, in reduced models this part is actually related to the diffusion part. In reduced models, several intermediate stages and species are lumped together or “adiabatically eliminated”, say by asymptotic methods. In classical approaches, this is typically done for well-mixed reaction (see e.g. the review^14^), whereas those intermediate species may, and often are, diffusive. Reduction in the full reaction-diffusion context, with account of different diffusivities of the eliminated intermediate reagents is not often done; however see ref. 15 for an example. That example is for an ecological model, but the mathematics involved is equally applicable for chemical reactions. That example shows, firstly, that effective diffusion coefficients occurring after the elimination are significantly different from those before (in particular, cross-diffusion terms appear where have been none originally) and, secondly, that these effective diffusion coefficients depend on the reaction kinetics linking the eliminated species with the main species — which is the place through which the remoteness from the thermodynamic equilibrium creeps right into the diffusion matrix. Another example, more specifically about chemical systems, is the complex Ginzburg-Landau equation, which is in fact a reaction-diffusion system where the diffusion matrix has a complex pair of eigenvalues, but it can be obtained by asymptotic reduction from a reaction-diffusion system with a diagonal diffusion matrix; see e.g. [ref. 16, Appendix B].

In this paper, we illustrate how the fast-slow analysis (adiabatic elimination of fast variables) in the spatially-extended context can be used to reconstruct full (fuller) reaction-diffusion systems with purely self-diffusing reagents, from reduced reaction-diffusion systems with cross-diffusion. We apply this procedure to produce examples of self-diffusion systems that manifest quasi-soliton solutions. Since the fast-slow analysis is an asymptotic procedure, i.e. is formally valid in certain parametric limits but has to be applied for fixed parameter values, it inherently has limited accuracy. The procedure we exploit here is valid when the spatial scale of the solutions is large compared to the diffusion length of the fast species, and the reduced equations become non-local when this is not fulfilled. To relax this restriction, we suggest a euristic procedure which enhances the accuracy of the reduction/reconstruction for particular wave-like solutions.

The structure of the paper is as follows. In section “Methods: Fast-slow reduction for reaction-diffusion systems” we discuss the process of adiabatic elimination of a fast reacting species in the spatially-extended context, and show how the diffusivity matrix may be affected by that elimination. In section “Results” we present a simple application of this theory: namely, by “working backwards” the adiabatic elimination, we construct a three-component reaction-diffusion system with self-diffusion only, which corresponds to a two-component system with cross-diffusion and quasi-soliton solutions of various kinds. We conclude by discussing possible implications of our findings for future research.

## Methods

### Fast-slow reduction for reaction-diffusion systems

Our asymptotic argument is based on two assumptions. Firstly, we consider a reaction-diffusion system, which has a fast and a slow subsystem,


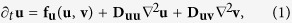



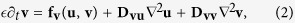


where 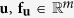, 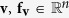, 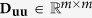, 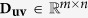, 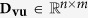, 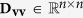, and the parameter 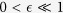 represents the time separation between the slow subsystem **u** and the fast subsystem **v**. We assume that the fast subsystem (2) has a unique globally stable equilibrium for **v** = **g**(**u**) at any fixed **u** of interest,


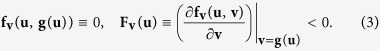


The second assumption, to be clarified and formalized later, is that the solutions of interest are sufficiently smooth in space.

Linearizing equation (2) around the stable equilibrium,


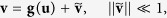


and considering the limit 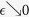, we have


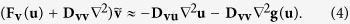


At this point we see that our solutions should be so smooth that ∇^2^**u** is small and therefore the resulting 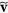 is small, justifying the linearization made. Formally, we set 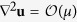, 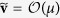 where *μ* is a (symbolical) small parameter. Then we can formally resolve equation (4), keeping terms up to and including 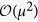, to get


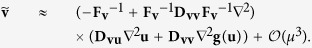


Substitution of the thus found **v** into equation (1) leads to


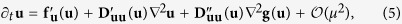


where 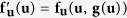, **F**_**u**_ = ∂_**v**_**f**_**u**_(**u**, **v**)|_**v**=**g**(**u**)_, and


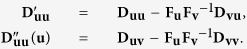


We see that the diffusion in the reduced system (5) is not only different from the full system, but is typically nonlinear even if the original diffusion was fully linear.

In the system (5) we have dropped 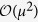 terms as they are complicated and will not be needed in this paper in the general form. In the special case when the fast subsystem is linear and is linked to the slow system in a linear way, the situation simplifies. We then have **g**(**u**) = **Gu**, **G** = const, and


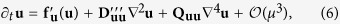


were


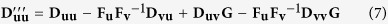


and


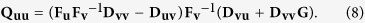


The second-order approximation (6) contains a biharmonic operator. We can approximately replace it with a reaction-diffusion system for solutions which are approximately oscillatory in space with one dominant wavenumber *k*, so that


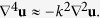


Then we have


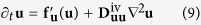


where 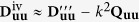. If the solutions of interest, or their Laplacians, are not approximate solutions of the Helmholtz equation, this second-order approximation is clearly only a euristic.

The direct transformation from system (1, 2) veq) to system (5) or (9), and its inverse, can be used for searching for nontrivial regimes in the full system, based on the existing experience of nontrivial regimes in the analogues of the reduced systems:

1. Do parametric search in the reduced system, which has fewer parameters and often is easier to compute, as it is less stiff;
2. Once an interesting regime is found, estimate the dominant wavenumber *k* for it, say as the maximum of the power spectrum of the spatial profile;
3. Use the inverse transformation to obtain parameters for the full system corresponding to the found parameters of the reduced system;
4. See what solutions the full system with these parameters will produce.

## Results

### Direct transform (reduction)

As a simple example, we now consider a three-component extension of a two-component system with nonlinear kinetics, where a third component is added, which has fast linear kinetics and linked to the other components in a linear way:


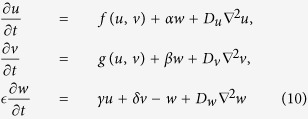


In terms of equations (1, 2), this means


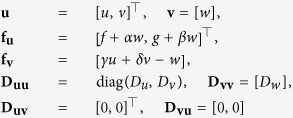


which leads to the reduced system (9) in the form


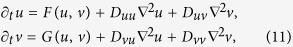


where


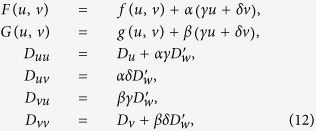


and


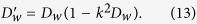


It is easy to see that the resulting diffusivity matrix


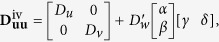


depends on the reaction kinetics parameters and does not have to have real eigenvalues or even be positive definite (although the utility of the reduced model is of course stipulated by the well-posedness of its Cauchy problems).

### Inverse transform (reconstruction)

Equation (12) allow to determine parameters of the two-component system (11) from the given system (10). For the study presented later, we need also to be able to do the opposite: assuming that a two-component system (11) is known, and is known to be a reduction of a three-component system (10), to reconstruct the parameters of that three-component system. One obvious restriction is that it is not possible to reconstruct the value of parameter 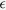 since the known system (11) corresponds to the zero limit of that parameter. However, the non-uniqueness of the inverse transform is even stronger than that, and there are infinitely many ways to choose parameters of (10), which would correspond to the same (11), even disregarding the variety of 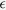. For instance, we can also arbitrarily fix values of *γ*, *D*_*w*_, *D*_*v*_, and for given parameters of (11) (and given dominant wavenumber *k*, if using the euristic second-order approximation), obtain the remaining parameters of (10) via


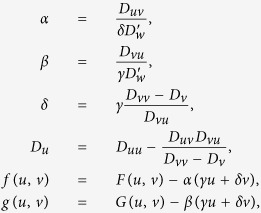


and 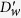 is still related to *D*_*w*_ via equation (13). The only restriction is that the resulting reconstructed system should be physically reasonable. This, in particular, requires that *D*_*u*_ ≥ 0. The latter will be satified if *D*_*v*_ is chosen so that either *D*_*v*_ ≤ *D*_*uu*_*D*_*vv*_/(*D*_*u*_ + *D*_*uu*_) or *D*_*v*_ ≥ *D*_*vv*_.

### Quasi-solitons in reduced system with FitzHugh-Nagumo kinetic

**Figure 1:**
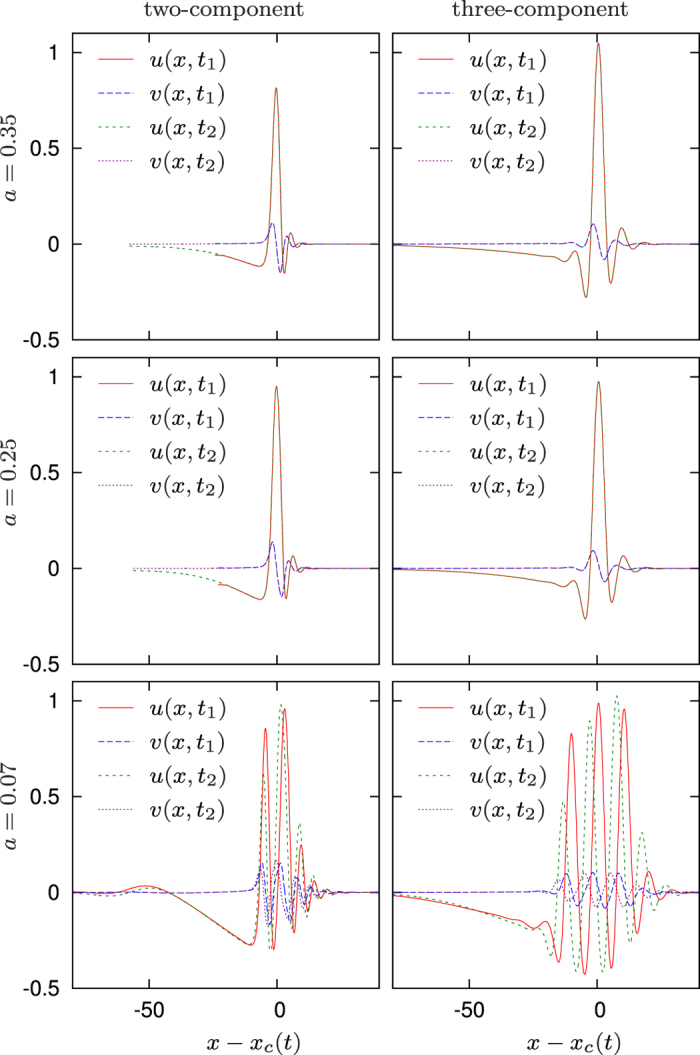
Selected profiles of quasi-soliton solutions for the FitzHugh-Nagumo kinetics, in co-moving frames of reference. Left column: two-component model with self- and cross-diffusion. Right column: corresponding reconstructed three-component model with self-diffusion only. The values of the parameter *a* are given to the left of the panels, for other parameters see the text. The coordinate *x* − *x*_*c*_ is in the comoving frame of reference, see text for detail. For each case, two profiles are shown at the moments *t* = *t*_1_ and *t* = *t*_2_ separated by an interval *t*_2_ − *t*_1_ = 150; however, the consecutive profiles are only different for *a* = 0.07 case, as in the other two cases, the waves have steady shapes (see also Apple QuickTime movies corresponding to these regimes in Supplementary information).

**Figure 2:**
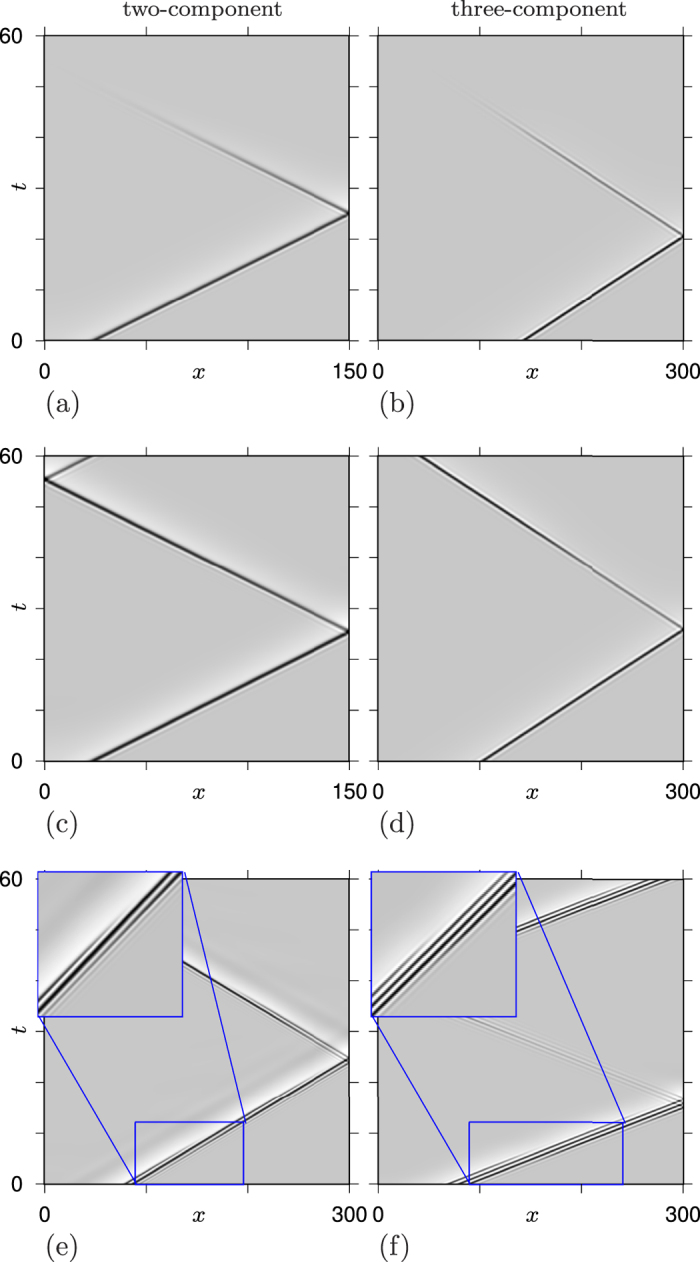
Density plots of the solutions represented in Fig. 1, in particular: (**a,b**) correspond to *a* = 0.35, (**c,d**) correspond to *a* = 0.25, (**e,f**) correspond to *a* = 0.07, left column represents two-component model with cross-diffusion, right column the corresponding reconstructed three-component model with self-diffusion only. White corresponds to *u* = −0.3, black corresponds to *u* = 1.1. The insets in panels (**e,f**) (designated by solid blue lines) show selected fragments of the density plots magnified, to reveal the fine structure of group quasi-solitons (see also Apple QuickTime movies corresponding to these regimes in Supplementary information).

Numerical illustrations of some nontrivial regimes obtained in the two-component system, and corresponding nontrivial regimes found in the reconstructed three-component system are presented in Figs 1 and 2. These are for the two-component system (11) with the FitzHigh-Nagumo kinetics, taken in the form


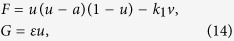


and the corresponding three-component system (10). We used the parameter values *ε* = 0.01, *k*_1_ = 10, 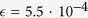, *D*_*uu*_ = *D*_*vv*_ = 0.025, *D*_*uv*_ = 1, *D*_*vu*_ = −1, *γ* = 30, *D*_*v*_ = 0.001, *D*_*w*_ = 0.04, and a selection of values of *a* as shown to the left of the panels in Fig. 1. The dominant wavenumbers *k* for the inverse transform were obtained as the position of the maximum of the power spectrum of the signal *z*(*x*) = *u*(*x*, *t*) + *iv*(*x*, *t*) of a selected solution (*u*, *v*) of the two-component system, taken at a selected fixed *t*. Specifically, we had *k* = 0.864 for *a* = 0.07, *k* = 0.879 for *a* = 0.25 and *k* = 0.942 for *a* = 0.35. For simulations, we used the same numerical methods as those described in refs 2 and 12. Computations were done using second-order space differencing on the uniform grid with space step Δ_*x*_ = 0.1. Time differencing was first-order, explicit in the reaction terms and implicit in the cross-diffusion terms, with time step Δ_*t*_ = 2.5 ⋅ 10^−4^ for the two-component system, and fully explicit with time step Δ_*t*_ = 2 ⋅ 10^−5^ for the three-component system. The space interval was *x* ∈ [0, *L*], where *L* was different in different simulations, as shown in *x*-axis labels in Fig. 2. Initial conditions were set as *u*(*x*, 0) = *u*_*s*_H(*σ* − *x*), *v*(*x*, 0) = 0, *w*(*x*, 0) = 0 to initiate a wave starting from the left end of the domain. Here H() is the Heaviside function, the wave seed amplitude was typically chosen as *u*_*s*_ = 2 and length as *σ* = 4. To simulate propagation “on an infinite line”, *L* = ∞, for Fig. 1, we instantanously translated the solution by *δx*_2_ = 20 away from the boundary each time the pulse, as measured at the level *u* = 0.1, approached the boundary to a distance smaller than *δx*_1_ = 40, and filled in the new interval of *x* values by extending the *u*, *v* and *w* variables at constant levels. The profiles in Fig. 1 are drawn in moving frames of reference; the “co-moving” space coordinate is defined as *x* − *x*_*c*_, where *x*_*c*_ = *x*_*c*_(*t*) is the center of mass of *v*^2^ at the time moment *t*, that is, 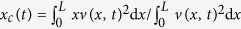.

For *a* = 0.35 and *a* = 0.25, both 2-component and 3-component systems support propagation of a solitary pulse with oscillating front and monotonous back. These shapes are steady: as one can see in the top four panels of Fig. 1, in the moving frame of reference, the solutions do not depend on time. The difference is that for *a* = 0.35, the pulses annihilate upon collision with a bondary, — or, rather, reflect but lose their magnitude, cannot recover and eventually decay. Whereas for *a* = 0.25, the pulses in both systems successfully reflect from boundaries. One can see in Fig. 2(c) the reflection from the right boundary, followed by reflection from the left boundary; the process repeats after that (not shown). The behaviour in the three-component variant of the system is similar, although the second reflection is not shown in panel (d). We emphasize here that the established shape, speed and amplitude of propagating pulses depend only on the parameters of the model, but not on initial conditions, as long as the initial condition is such that a propagating pulse is initiated. For instance, the profiles of the solutions obtained for *σ* = 2, *σ* = 8 and *σ* = 12 were indistinguishable from those shown in Fig. 1. These regimes are similar to those described in ref. 3.

The behaviour for *a* = 0.07 is different: the shape of the pulse changes as it propagates. These are “envelope quasisolitons”, similar to those previously reported in in refs 2 and 12, and can be described as modulated high-frequency waves with the envelope in the form of a solitary wave, where the speed of the high-frequency waves (the “phase velocity”) is different from the speed of the envelope (the “group velocity”), thus the change in shape. Note that this behaviour is similar to solitons in the nonlinear Schrödinger equation (NLS)^17^, both in terms of the varying shape and the reflection from boundary, with the difference that here the system is dissipative so here again there are unique and stable amplitude and envelope shape, with corresponding group and phase velocities, which are determined by the parameters of the system but do not depend on initial conditions, as long as a propagating wave is initiated.

Since the speed and shape of quasi-solitons are typically fixed, the reflections and collisions, when they happen, are always quasi-elastic, if one is to use the terminology from the conservative wave theory. That is, the properties of the solitons fully recover after collisions, even though, as can be seen in Fig. 2, it make take some time. On the other hand, there are examples of quasi-solitons models in which the unique shape of quasi-solitons takes a very long time to establish, so at short time intervals, one may consider a one-parametric family of quasi-soliton solutions: in ref. 18, these are solutions differing in their “thickness”, i.e. distance between the front and the back. The rules of collision there are more complicated, e.g. there could be “completely inelastic” collisions, where of the two colliding waves one survives and the other annihilates.

Finally, we emphasize that the reflection from boundaries shown in Fig. 2 is for homogeneous Neumann boundary condition. Replacing those with e.g. homogeneous Dirichlet boundary conditions makes reflection much less likely. In particular, it is not observed in any of the six cases shown in Figs 1 and 2, although can be observed for other parameter values, for instance for *a* = 0.03, and even then the reflected wave takes much longer to fully recover. In this aspect, the quasi-solitons are also different from the true solitons, e.g. in the NLS. A formal way to understand this difference is to observe that NLS is symmetric with respect to inversion of the sign of its complex field. Hence one can arrange two identical but counter-propagating solitons on an infinite line so that their nonlinear superposition at a certain point remains exactly zero at all times. Then replacing the problem with the one at half-line and zero boundary condition at that point will yield a solution in the form of a soliton reflecting from the boundary with a 180° change of phase. This construction does not work for the FitzHugh-Nagumo kinetics (14) which is not invariant with respect to the inversion (*u*, *v*) → (−*u*, −*v*), unless *a* = −1, but in the latter case the system is no longer excitable. A more intuitive way to explain this is: for the pulse way to propagate, the *u* field must exceed the threshold *a*, and the boundary condition *u* = 0 makes it much more difficult for the reflected wave to satisfy this.

## Discussion

It has been traditionally believed that a definitive property of waves in excitable media is that they annihilate when collided. Although soliton-like interaction was observed in some reaction-diffusion systems with excitable kinetics, both in numerical simulations^19^,^20^ and in experiments^21^,^22^, solitons are mostly studied in fully integrable systems (KdV, sin-Gordon, nonlinear Schrödinger). Perhaps the main reason of this view on excitable media was that soliton-like interactions were always limited to narrow parameter ranges close to the boundaries between excitable and oscillatory (limit cycle) regimes of the reaction kinetics. A crucial role in the change of the attitude to excitable media as a source of solitons was played by experimental and theoretical works by Vanag and Epstein^6^,^23^,^24^,^25^. They have demonstrated reaction-diffusion systems with soliton-like interaction of waves, and also spontaneous formation of wave packets. At the same time, we have shown that in excitable systems with cross-diffusion, the soliton-like behaviour of waves can be quite typical, including solutions similar to group (envelope) solitons^12^. These works resonate with Vanag and Epstein’s reports of cross-diffusion in the chemical system BZ-AOT^6^. In the present work, we have demonstrated that quasi-solitons, including group (envelope) quasi-solitons, can observed in reaction-diffusion systems with self-diffusion only. This has been found with the help of two-component systems with effective cross-diffusion, which are obtained by semi-rigorous adiabatric reduction of a multicomonent reaction-diffusion system with self-diffusion only. Adiabatic elimination of fast fields, which gives rise to nontrivial dissipative terms, that, for physical reasons, cannot exist in the straightforward form, can be observed in various physical settings. For instance, in nonlinear optics, adiabatic elimination of the acoustic field gives rise to an extra term in the NLS equation for the optical field, that is similar to stimulated Raman scattering which cannot appear in that equation directly, thus dubbed “pseudo-stimulated-Raman-scattering”^26^,^27^,^28^. By the analogy with that result, the effective cross-diffusion we described here could be called “pseudo-cross-diffusion”. For the purposes of the present communication, an important feature of the effective cross-diffusion is that the resulting diffusivity matrix is *not* constraint by the thermodynamic restrictions of symmetry and positive definiteness. We believe that application of such reduction, accounting for the emergence of effective cross-diffusion, may lead to finding new interesting regimes in systems that have traditionally been studied without cross-diffusion, e.g. Brusselator^29^ and Oregonator^30^,^31^, which remains an interesting direction for further study.

## Additional Information

**How to cite this article**: Biktashev, V. N. and Tsyganov, M. A. Quasisolitons in self-diffusive excitable systems, or Why asymmetric diffusivity obeys the Second Law. *Sci. Rep.* **6**, 30879; doi: 10.1038/srep30879 (2016).

## Supplementary Material

Supplementary Information

Supplementary Movies

Supplementary Movies

Supplementary Movies

Supplementary Movies

Supplementary Movies

Supplementary Movies
