# Supplementary material for: Quasisolitons in self-diffusive excitable systems, or Why asymmetric diffusivity obeys the Second Law
Source: Sci Rep. 2016 Aug 5;6:30879. doi: 10.1038/srep30879 (PMC4974638; doi:10.1038/srep30879)
Supplement: Supplementary Information [file srep30879-s1.pdf]

Quasisolitons in self-diffusive excitable systems, or Why asymmetric diffusivity obeys the Second Law

V. N. Biktashev[1], M. A. Tsyganov[2]
[1] College of Engineering, Mathematics and Physical Sciences, University of Exeter, Exeter EX4 4QF, UK, and EPSRC Centre for Predictive Modelling in Healthcare, University of Exeter, Exeter, EX4 4QJ, UK
[2] Institute of Theoretical and Experimental Biophysics, Pushchino, Moscow Region, 142290, Russia

Supplementary videos

The six computer-generated videos show the moving graphs of the six solutions illustrated in figures 1 and 2 of the main text, corresponding respectively to:

fhn2-07.mov : two-component system, $a = 0.07$;

fhn2-25.mov : two-component system, $a = 0.25$;

fhn2-35.mov : two-component system, $a = 0.35$;

fhn3-07.mov : three-component system, $a = 0.07$;

fhn3-25.mov : three-component system, $a = 0.25$;

fhn3-35.mov : three-component system, $a = 0.35$.

In all movies, red lines are graphs of the u component and blue lines are graphs of the v component. For the three-component systems, we also show the graphs of the w component as green lines.
